# Association Between First-Trimester Vitamin D Levels and Gestational Diabetes Mellitus: A Prospective Observational Study From South India

**DOI:** 10.7759/cureus.95365

**Published:** 2025-10-25

**Authors:** Harshinee N, Preethi B, Jasmine Kavitha Washington

**Affiliations:** 1 Obstetrics and Gynaecology, Sree Balaji Medical College and Hospital, Chennai, IND

**Keywords:** first-trimester pregnancy, gestational diabetes mellitus (gdm), prenatal maternal screening, roc curve analysis, vitamin d deficiency

## Abstract

Background: Vitamin D deficiency and gestational diabetes mellitus (GDM) are two overlapping public health concerns that are increasingly prevalent among pregnant women. This study was designed to assess whether serum vitamin D deficiency in the first trimester is associated with the development of GDM in an Indian antenatal population. This study aimed to evaluate the association between first-trimester serum 25-hydroxyvitamin D [25(OH)D] levels and subsequent development of GDM.

Methods: This prospective observational study was conducted over 18 months in a tertiary care center in South India. Eighty-seven singleton pregnant women in their first trimester (<13 weeks of gestation) were recruited. Exclusion criteria included pre-gestational diabetes and a previous history of GDM. Serum 25(OH)D was measured using chemiluminescence immunoassay (CLIA). The oral glucose challenge test was utilized for GDM screening at 24-28 weeks and again at 32-34 weeks. Statistical analyses included logistic regression and an ROC curve to identify predictive thresholds.

Results: Vitamin D deficiency (<20 ng/mL) was detected in 60.9% of participants. GDM occurred in 30 women, with a significant association noted between vitamin D deficiency and GDM (p = 0.001). Logistic regression revealed that vitamin D deficiency conferred a fivefold increased risk of GDM (aOR: 5.03; 95% CI: 2.12-11.94). ROC analysis demonstrated high predictive accuracy (AUC = 0.94), with <19.5 ng/mL identified as the optimal threshold (sensitivity: 88.2%, specificity: 91.3%).

Conclusion: First-trimester vitamin D deficiency was significantly associated with increased risk of developing GDM later in pregnancy. These findings support routine early pregnancy screening for vitamin D and suggest potential benefits of timely nutritional interventions in high-risk populations.

## Introduction

Gestational diabetes mellitus (GDM) represents a growing global public health concern due to its short- and long-term health implications for both the mother and fetus. Defined as glucose intolerance with onset or first recognition during pregnancy, GDM is associated with numerous maternal complications, including hypertension, preeclampsia, and increased rates of cesarean delivery, as well as heightened lifetime risk of type 2 diabetes mellitus (T2DM). For the fetus, adverse outcomes range from macrosomia and neonatal hypoglycemia to respiratory distress syndrome and long-term metabolic disorders. The global prevalence of GDM has been estimated at approximately 14%, though this figure varies by region owing to differences in diagnostic criteria, genetic susceptibility, and nutritional and lifestyle practices [1]. Importantly, the escalating prevalence is closely linked to rising maternal obesity, sedentary behavior, and advanced maternal age, underscoring the urgent need for effective preventive and predictive strategies.

In recent years, research has expanded beyond conventional risk factors to explore the contribution of micronutrients, particularly vitamin D, to the pathophysiology of GDM. Traditionally recognized for its role in calcium homeostasis and bone metabolism, vitamin D is now understood to exert broader biological effects, including modulation of immune responses, regulation of insulin secretion, and maintenance of glucose homeostasis [1]. Deficiency of vitamin D during pregnancy has thus been hypothesized as a potential contributor to impaired glucose tolerance. The underlying mechanisms are biologically plausible: vitamin D receptors are expressed in pancreatic β-cells, where active metabolites influence insulin gene transcription and secretion. In addition, vitamin D reduces systemic inflammation and improves insulin sensitivity, both of which are critical pathways implicated in the development of GDM [1].

A growing body of evidence supports the link between vitamin D deficiency and adverse pregnancy outcomes. Agarwal et al. synthesized findings from multiple studies, reporting that low maternal vitamin D levels are associated with preeclampsia, low birth weight, and a higher incidence of GDM [1]. They emphasized that vitamin D plays a dual role, supporting fetal skeletal development while also regulating placental function and maternal glucose metabolism. Consistent with this, a systematic review and meta-analysis by Wei et al. demonstrated that maternal deficiency of 25-hydroxyvitamin D significantly increases the risk of preterm birth, small-for-gestational-age infants, and GDM, with associations persisting even after adjusting for confounders such as maternal age and BMI [2].

Global prevalence data highlight the scale of this problem. Reverzaniet al. reported that over 60% of pregnant women worldwide have insufficient vitamin D levels, with particularly high prevalence in South Asia and the Middle East [3]. Their meta-analysis found consistent associations between low vitamin D and higher GDM risk, especially among populations with darker skin pigmentation, limited sun exposure, or restrictive clothing practices. Similarly, Poel et al., in one of the earliest meta-analyses focused exclusively on vitamin D and GDM, found that deficiency was associated with a 61% higher risk of developing GDM, strengthening the case for standardized screening during pregnancy [4]. Extending this evidence, Zhang et al. confirmed a dose-response relationship, with progressively lower serum vitamin D concentrations corresponding to increased GDM risk [5].

Despite robust observational evidence, interventional studies have yielded mixed results. Pérez-López et al., in their systematic review of randomized controlled trials (RCTs), reported that vitamin D supplementation improved maternal serum levels and reduced the risk of preeclampsia and low birth weight, though its effects on GDM incidence varied considerably [6]. These inconsistencies may stem from heterogeneity in study design, supplementation regimens, and baseline vitamin D status. Nonetheless, supplementation during pregnancy remains safe and potentially beneficial for multiple maternal and neonatal outcomes [6].

Prospective cohort studies have further clarified the temporal association between vitamin D status and GDM. Burris et al. showed that first-trimester vitamin D deficiency (<25 nmol/L) doubled the risk of developing GDM in a large, ethnically diverse cohort, even after controlling for BMI, age, and race/ethnicity [7]. Fatima et al. similarly demonstrated that women with deficient vitamin D levels in early pregnancy had a significantly higher incidence of GDM, with regression models confirming vitamin D as an independent predictor [8]. Complementing these findings, Lacroix et al. in Canada observed that lower vitamin D levels in the first trimester were linked to higher plasma glucose at 24-28 weeks and increased GDM risk, with threshold effects evident at 25(OH)D <50 nmol/L [9]. Makgoba et al. further proposed a mechanistic link, suggesting that vitamin D regulates placental inflammation and insulin resistance, thereby influencing GDM development [10].

The Indian context warrants special attention, given the dual burden of high GDM prevalence (15-20% in urban populations) and widespread vitamin D deficiency affecting up to 80% of pregnant women. Contributing factors include limited sun exposure due to cultural clothing norms, poor dietary intake, high parity, and rising obesity rates. These overlapping risk profiles create an urgent need for region-specific research to clarify the role of vitamin D in GDM pathogenesis and to inform locally relevant guidelines.

The present study aims to address this critical evidence gap by prospectively evaluating the association between first-trimester vitamin D levels and subsequent development of GDM, diagnosed using the DIPSI criteria. By combining baseline biochemical measurements with longitudinal follow-up, this study seeks to establish whether early maternal vitamin D status can serve as a predictor of GDM. Findings from this research could have significant implications for early screening, targeted intervention, and prevention strategies, particularly in resource-limited settings such as India, where the burden of both GDM and vitamin D deficiency is alarmingly high.

## Materials and methods

Study design and setting

This was a prospective observational study conducted over a period of 18 months in the Department of Obstetrics and Gynaecology at Sree Balaji Medical College and Hospital, Chennai, South India. As a tertiary care center, the institution caters to a heterogeneous population drawn from both urban and semi-urban areas. This diversity provided an ideal setting to investigate the prevalence of vitamin D deficiency during pregnancy and its association with GDM. The study was designed to follow women longitudinally from early pregnancy through the antenatal period, enabling the evaluation of first-trimester vitamin D status in relation to subsequent glucose intolerance.

Study population

The study population comprised antenatal women attending the outpatient department (OPD) for their first antenatal visit. Women were screened consecutively, and those meeting eligibility criteria were invited to participate after being provided with detailed information regarding the objectives, procedures, and implications of the study. Written informed consent was obtained in the local language prior to enrollment.

Inclusion and exclusion criteria

Eligibility was restricted to women with singleton pregnancies confirmed by ultrasonography to be within the first trimester, defined as gestational age less than 13 weeks and six days. This cutoff was selected to ensure that maternal vitamin D levels reflected early pregnancy status, prior to the metabolic alterations of mid and late gestation. Exclusion criteria were carefully applied to minimize confounding variables. Women with known pre-gestational diabetes mellitus or a previous history of GDM were excluded, as these conditions could independently alter glucose metabolism and bias the assessment of vitamin D’s role. Additional exclusions included women with multiple gestations, known endocrinopathies such as thyroid or adrenal disorders, and those unwilling to undergo serial follow-up visits.

Sample size estimation

The minimum required sample size was estimated based on published literature. Zhang et al. [5] reported that vitamin D deficiency increased the risk of developing GDM nearly fivefold. Assuming a prevalence of vitamin D deficiency of approximately 60% among pregnant women, with 80% power and a 5% level of significance, the required sample size was calculated to be 87 participants. The calculation was performed using R statistical software (R Foundation for Statistical Computing, Vienna, Austria), with a design effect of 1.0. This ensured that the study had adequate power to detect a meaningful association between vitamin D levels and GDM risk.

Sampling technique

A convenient sampling technique was adopted, whereby eligible women attending the antenatal OPD during the study period were consecutively approached. Although convenience sampling may carry limitations in generalizability, it allowed for practical recruitment within the available timeframe and resources while ensuring that a sufficient number of participants were included.

Data collection procedures

Data collection was carried out using a structured, pre-validated case record form. At baseline, detailed demographic and clinical information was obtained, including maternal age, educational status, socioeconomic class using the Modified BG Prasad Scale [11], and obstetric history. Lifestyle factors such as dietary intake of vitamin D-rich foods, duration of daily sun exposure, and level of physical activity were also recorded, as these variables are known to influence vitamin D status.

Anthropometric measurements were obtained by trained personnel using calibrated instruments. Maternal height was measured using a stadiometer, and weight was assessed using a digital weighing scale. BMI was calculated as weight in kilograms divided by the square of height in meters (kg/m²). These measurements were taken at the time of enrollment to establish baseline nutritional status.

Biochemical analysis

Venous blood samples were collected during the first trimester visit. Serum concentrations of 25-hydroxyvitamin D [25(OH)D], the primary circulating form of vitamin D, were estimated using a fully automated chemiluminescent immunoassay (CLIA) technique in the central biochemistry laboratory of the institution. To ensure reliability of results, samples were centrifuged within one hour of collection and analyzed on the same day. The Endocrine Society’s clinical practice guidelines were used to categorize vitamin D status: deficiency (<20 ng/mL), insufficiency (20-30 ng/mL), and sufficiency (>30 ng/mL).

Diagnosis of gestational diabetes mellitus

Screening for GDM was performed according to the Diabetes in Pregnancy Study Group of India (DIPSI) guidelines [12], which are widely utilized in the Indian context due to their simplicity and feasibility. All enrolled participants underwent a 75-gram oral glucose challenge test at 24-28 weeks of gestation, irrespective of their last meal timing. A venous blood sample was obtained two hours after the glucose load, and plasma glucose was measured. A value of ≥140 mg/dL was considered diagnostic of GDM. Women with normal results were retested at 32-34 weeks to account for late-onset GDM. The DIPSI criterion, being a single-step, non-fasting test, was particularly suited for use in a busy tertiary care hospital setting and minimized participant inconvenience.

Ethical considerations

The study protocol was reviewed and approved by the Institutional Ethics Committee of Sree Balaji Medical College and Hospital prior to commencement (Ref. No: 002/SBMCH/IHEC/2023/2035). Participation was entirely voluntary, and all women were provided with written informed consent forms in their local language. Confidentiality of participant data was maintained at all stages, and results were used solely for academic purposes. The study adhered to the principles of the Declaration of Helsinki, ensuring respect for autonomy, beneficence, and non-maleficence.

Statistical analysis

Data were entered into Microsoft Excel and subsequently analyzed using IBM SPSS Statistics for Windows, Version 26 (Released 2019; IBM Corp., Armonk, New York, United States). Continuous variables such as maternal age, BMI, and serum vitamin D levels were tested for normality. Normally distributed variables were expressed as mean ± standard deviation, while skewed data were summarized as median with interquartile range. Independent sample t-tests were used to compare mean vitamin D levels between GDM and non-GDM groups when data were normally distributed, whereas the Mann-Whitney U test was employed for non-parametric comparisons.

Categorical variables, such as vitamin D deficiency status and GDM occurrence, were expressed as frequencies and percentages. Associations between categorical variables were assessed using the chi-square test or Fisher’s exact test where appropriate. Pearson or Spearman correlation coefficients were calculated to evaluate the relationship between vitamin D levels and continuous maternal parameters such as BMI, age, and plasma glucose.

To determine whether vitamin D deficiency independently predicted GDM, binary logistic regression analysis was performed. Potential confounders including maternal age, BMI, and sunlight exposure were entered into the model. Adjusted odds ratios (aOR) with 95% confidence intervals (CIs) were reported. Receiver operating characteristic (ROC) curve analysis was carried out to assess the diagnostic accuracy of serum vitamin D levels in predicting GDM. The area under the ROC curve (AUC) was calculated to quantify discriminative ability. The optimal vitamin D cut-off value for identifying GDM was determined using Youden’s Index, and diagnostic indices including sensitivity, specificity, positive predictive value (PPV), and negative predictive value (NPV) were calculated. For all statistical tests, a two-sided p-value of <0.05 was considered statistically significant.

## Results

Among the 87 study participants, the majority were aged 31-35 years (37, 42.5%), followed by 26-30 years (25, 28.7%), while only two women (2.3%) were above 35 years. Participants' average age was 28.62 years, with a standard deviation of 4.79. Nearly half of the women were overweight (43, 49.4%), while 36 (41.4%) had normal BMI, and a small proportion were underweight (4, 4.6%) or obese (4, 4.6%). The mean BMI was 26.95, with a standard deviation of 3.49, suggesting overweight in the study population. More than half of the women were multigravida (52, 59.8%), with 35 (40.2%) being primigravida. In terms of socioeconomic status, the majority belonged to the lower middle (30, 34.5%) and upper middle (26, 29.9%) classes, while eight participants (9.2%) were from the lower class. With respect to physical activity, a sedentary lifestyle was most common (43, 49.4%), followed by moderate activity (26, 29.9%) and heavy work (18, 20.7%) (Table 1).

**Table 1:** Baseline Characteristics of the Study Participants (n = 87) #Modified BG Prasad Scale

Variable	Category	Frequency (n)	Percentage (%)
Age (years)	<20	7	8.04
	20–25	16	18.39
	26–30	25	28.73
	31–35	37	42.52
	36–40	2	2.29
BMI*	Underweight	4	4.59
	Normal	36	41.37
	Overweight	43	49.42
	Obese	4	4.59
Parity	Primigravida	35	40.22
	Multigravida	52	59.77
Socioeconomic status#	Upper	10	11.49
	Upper middle	26	29.88
	Lower middle	30	34.48
	Upper lower	13	14.94
	Lower	8	9.19
Physical activity	Sedentary work	43	49.40
	Moderate work	26	29.88
	Heavy work	18	20.68

**Figure 1:**
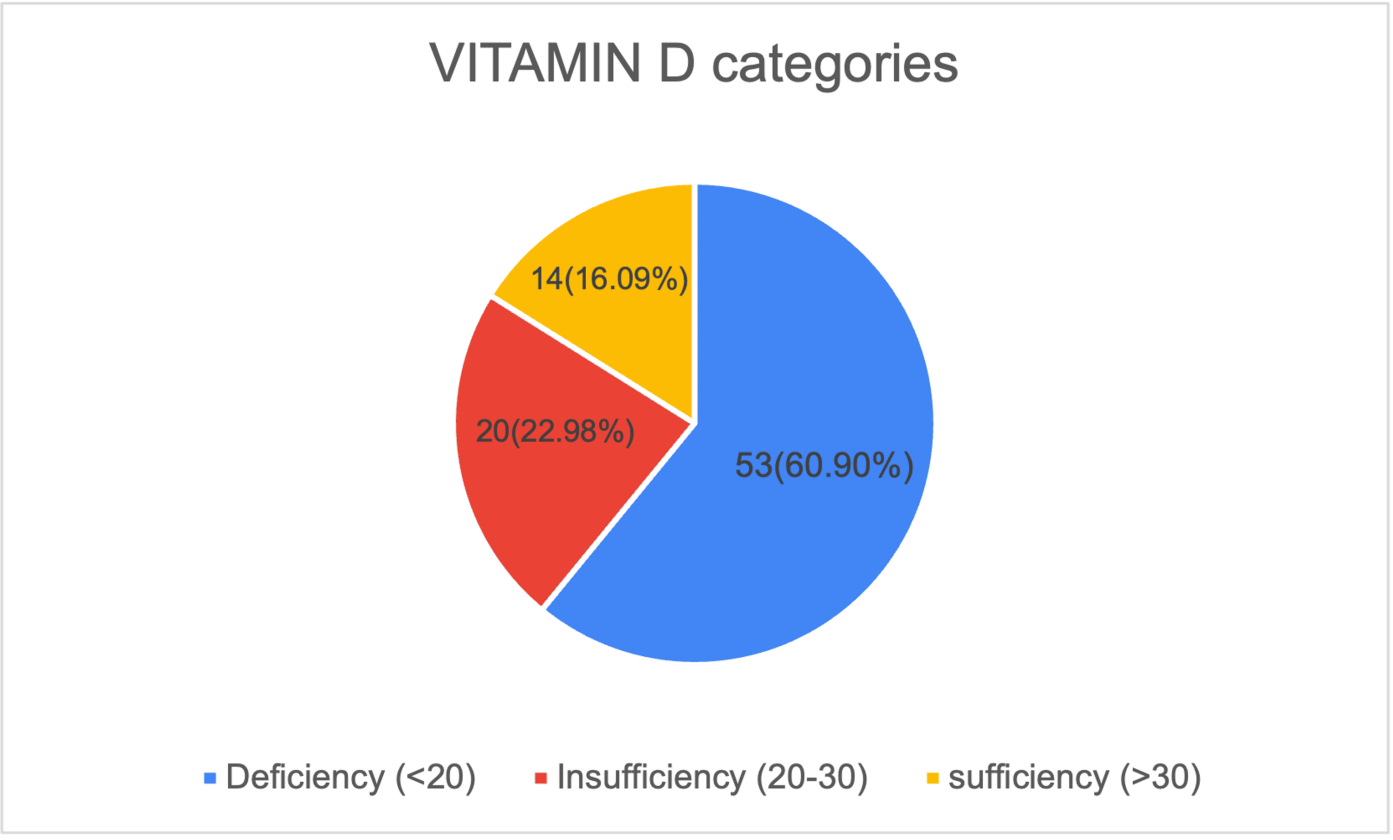
Descriptive Statistics of Vitamin D Levels among Study Participants The diagram shows the distribution of vitamin D levels in n(%) values.

Assessment of vitamin D status among the study participants revealed a high prevalence of deficiency. Out of 87 antenatal women, 53 (60.9%) were found to have vitamin D deficiency (<20 ng/mL), while 20 (23.0%) had insufficiency (20-30 ng/mL). Only 14 women (16.1%) had sufficient vitamin D levels (>30 ng/mL). This indicates that nearly four out of five participants had suboptimal vitamin D status during early pregnancy, underscoring a substantial burden of deficiency in this population (Figure 1).

Table 2 shows analysis that demonstrated a significant association between vitamin D status and GDM occurrence. Women with vitamin D deficiency showed the highest number of GDM cases (30 out of 53), while markedly fewer cases were observed in the insufficiency and sufficiency groups (three each). The chi-square test confirmed this association to be statistically significant (χ² = 13.09, p = 0.001). These findings highlight that vitamin D deficiency may substantially increase the risk of developing GDM during pregnancy. 

**Table 2:** Association between Vitamin D Categories and GDM Occurrence *Chi-square/Fisher's exact test p-value<0.05 statistically significant GDM: Gestational diabetes mellitus

Vitamin D Category	Yes (GDM)	No (GDM)	Chi-square Value	p-value
Deficiency (n = 53)	30	23	13.09*	0.001
Insufficiency (n = 20)	3	17		
Sufficiency (n = 14)	3	11		
Total	36	51		

**Figure 2:**
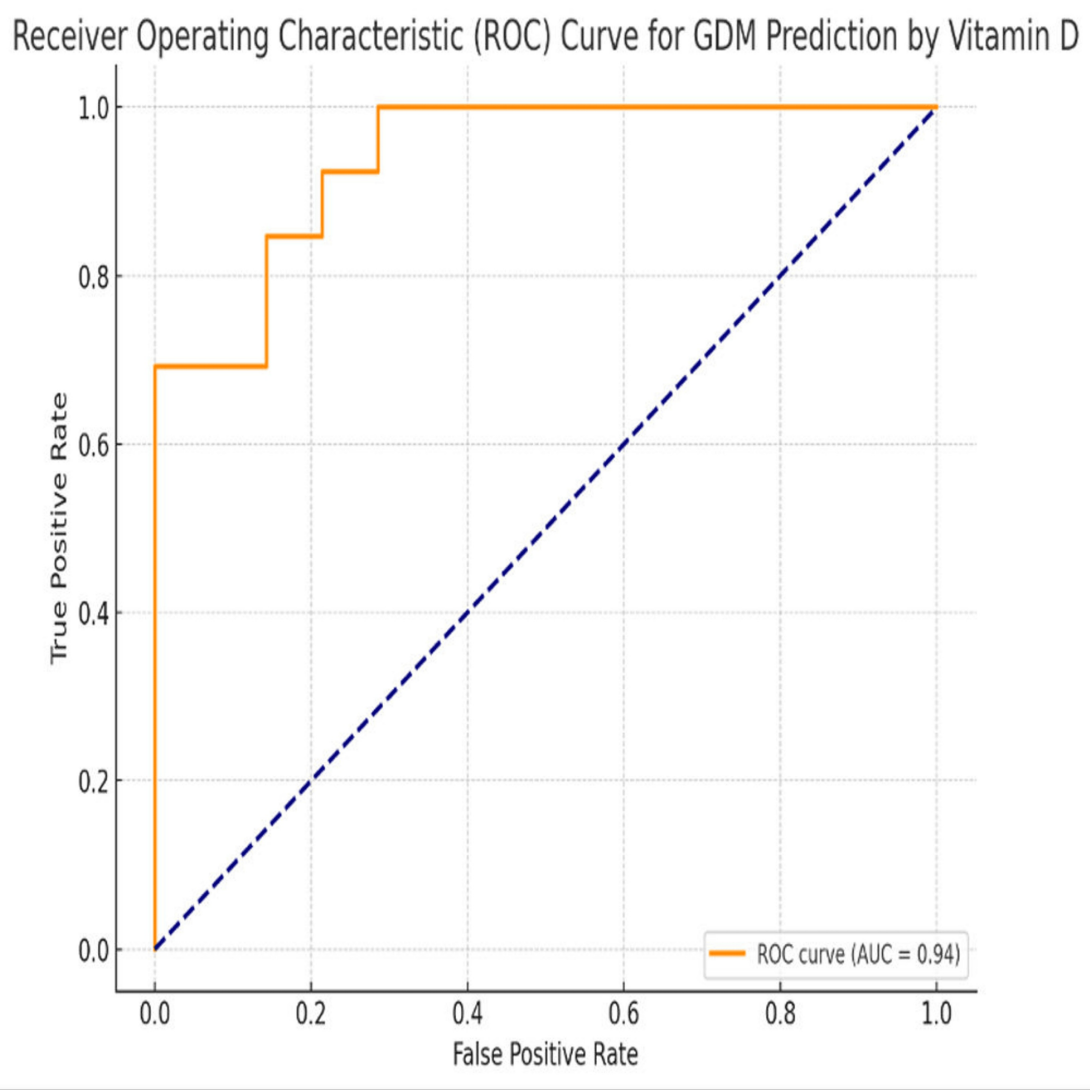
Receiver Operating Characteristic (ROC) Curve Analysis GDM: Gestational diabetes mellitus

Table 3 and Figure 2 show ROC curve analysis revealed excellent diagnostic accuracy of vitamin D levels in predicting GDM, with an AUC of 0.94 (95% CI: 0.89-0.99), indicating strong discriminatory power. The optimal cutoff value determined by the Youden Index was <19.5 ng/mL, yielding a sensitivity of 88.2% and specificity of 91.3%. The predictive values were also robust, with PPV of 83.3% and NPV of 93.7%. Furthermore, the likelihood ratios demonstrated strong clinical utility, with a positive likelihood ratio of 10.13 suggesting a markedly increased probability of GDM when the test is positive, while a negative likelihood ratio of 0.13 significantly lowered the probability when negative. The statistical significance of the ROC analysis (p < 0.001) reinforces the reliability of these findings.

**Table 3:** Receiver Operating Characteristic (ROC) Curve Analysis for Serum Vitamin D Status in GDM GDM: Gestational diabetes mellitus

Parameter	Value
Area Under the Curve (AUC)	0.9396
95% Confidence Interval (CI) for AUC	0.8912 – 0.9881
Optimal Cutoff Value (Youden Index)	<19.5 ng/mL
Sensitivity at Optimal Cutoff	88.2%
Specificity at Optimal Cutoff	91.3%
Positive Predictive Value (PPV)	83.3%
Negative Predictive Value (NPV)	93.7%
Likelihood Ratio (Positive)	10.13
Likelihood Ratio (Negative)	0.13
p-value for ROC Significance	<0.001

The correlation analysis revealed that serum vitamin D levels showed no significant association with age (r = -0.047, p > 0.05). However, a significant negative correlation was observed with both body mass index (BMI) (r = -0.327, p < 0.05) and GDM status (r = -0.318, p = 0.002). These findings suggest that higher BMI and the presence of GDM are linked with lower vitamin D levels, highlighting a potential role of vitamin D deficiency in the metabolic and clinical profile of affected individuals (Table 4).

**Table 4:** Correlation of Vitamin D Levels with Clinical and Biochemical Parameters Pearson correlation *p-value<0.05 statistically significant

Variable	Correlation Coefficient (r)	p-value*
Age	–0.047	>0.05
BMI	–0.327	<0.05
GDM Status	–0.318	0.002

## Discussion

This prospective observational study highlights a statistically significant association between maternal vitamin D deficiency in the first trimester and the subsequent development of GDM. Our findings add to the accumulating body of global evidence suggesting that inadequate serum 25-hydroxyvitamin D [25(OH)D] concentrations may play a key role in the pathogenesis of GDM.

In our cohort, vitamin D deficiency (<20 ng/mL) was observed in approximately two-thirds of participants (60.9%), and GDM was diagnosed in 30 of the 53 women with deficiency. The chi-square test demonstrated a highly significant association (p=0.001), and logistic regression analysis revealed an adjusted odds ratio (aOR) of 5.03. This indicates that vitamin D-deficient women were five times more likely to develop GDM compared to those with sufficient levels. The magnitude of this association is higher than the pooled estimate reported by Hu et al. in their meta-analysis of 20 studies, which identified a 1.53-fold increased risk of GDM with vitamin D deficiency [13]. The stronger effect size in our study may reflect unique contextual factors, such as regional differences in sunlight exposure, traditional clothing practices, dietary insufficiencies, and a high baseline prevalence of deficiency in South Indian women.

Zhao et al. also reported a robust association between maternal vitamin D deficiency and adverse pregnancy outcomes, including GDM, in their meta-analysis of prospective cohorts [14]. Their review emphasized the broader reproductive risks of hypovitaminosis D, such as preeclampsia and low birth weight. Our study not only supports their conclusions but also contributes trimester-specific data, underlining the predictive importance of early pregnancy vitamin D screening for identifying women at high risk of developing GDM.

The prevalence of deficiency in our population mirrors observations in other Asian cohorts. Song et al. investigated vitamin D status among pregnant women in Beijing and found that nearly 70% of participants had serum levels below 20 ng/mL, with clear associations with reduced neonatal size [15]. Although our study did not include neonatal anthropometry, the comparably high rates of maternal deficiency in both settings underscore a pressing need for region-specific nutritional guidelines and antenatal supplementation policies in Asia. The clinical relevance of supplementation has been highlighted by Palacios et al., who demonstrated in a meta-analysis that vitamin D supplementation improved maternal serum levels and modestly reduced the risks of GDM and preeclampsia [16]. Although our study was not interventional, the strength of the association between deficiency and GDM in our cohort strongly suggests that timely supplementation during early pregnancy may have preventive benefits.

The downstream benefits of maternal vitamin D sufficiency extend to offspring as well. A meta-analysis by Bi et al. reported that antenatal vitamin D supplementation reduced the incidence of small-for-gestational-age infants and respiratory infections in neonates [17]. While our study was limited to maternal outcomes, these findings provide additional justification for incorporating vitamin D screening and supplementation into routine antenatal care, given the potential for improving both maternal and fetal health outcomes.

A systematic review conducted by Harvey et al. under the UK Health Technology Assessment program concluded that vitamin D supplementation in pregnancy significantly reduced the risk of GDM and preterm birth, though heterogeneity across trials was a limiting factor [18]. Our observational results complement this review, demonstrating a significant inverse relationship between vitamin D concentrations and GDM risk. This reinforces the feasibility and cost-effectiveness of incorporating vitamin D assessment and supplementation into maternal health programs in high-burden populations.

Tabrizi et al. further substantiated this association in their meta-analysis, which reported a pooled odds ratio of 1.45 for GDM among vitamin D-deficient women, with consistent findings across diverse ethnic and geographic cohorts, including India [19]. Our results contribute to this body of evidence, providing contemporary Indian data with a notably higher risk estimate (aOR 5.03). This elevated risk may be attributable to synergistic risk factors in our cohort, including higher BMI, limited sun exposure due to cultural practices, and suboptimal dietary diversity. Nonetheless, it is important to note that not all Indian studies have reported similar associations. Farrant et al., in a large South Indian cohort, observed that although vitamin D insufficiency was common, it was not associated with GDM or neonatal size [20]. The discrepancy between their results and ours may be explained by differences in study design, gestational timing of blood sampling, diagnostic criteria for GDM, and residual confounding factors such as calcium or magnesium intake, which were not captured in our dataset.

Methodological variations in GDM diagnosis also warrant attention. Agarwal et al. proposed a simplified diagnostic algorithm based on fasting plasma glucose, which highlights how minor differences in diagnostic thresholds may influence case ascertainment and, consequently, associations with risk factors such as vitamin D status [21]. Our adherence to DIPSI criteria provides standardization, yet diagnostic heterogeneity across studies remains a potential source of variability in reported associations. Luo et al. reported findings similar to ours in an Indian cohort, showing that vitamin D deficiency was independently associated with increased risks of GDM, preterm labor, and cesarean section [22]. Their participants, like ours, tended to have higher BMI and lower physical activity, emphasizing the multifactorial interplay between metabolic and nutritional risk factors in Indian women. Our study expands on this by incorporating ROC curve analysis, which demonstrated excellent predictive accuracy of vitamin D levels for GDM (AUC = 0.94).

Additional mechanistic insights come from Deepa, who found that vitamin D deficiency in the first trimester was associated with elevated fasting and postprandial glucose levels in Indian women [23]. These biochemical findings parallel our results and suggest that hypovitaminosis D may contribute to impaired glucose tolerance through pathways involving pancreatic beta-cell function and insulin sensitivity. Evidence from neighboring South Asian populations also supports our findings. Muthukrishnan et al. reported significantly lower vitamin D levels among women with GDM compared to controls, with an odds ratio of 2.4 [24]. Although lower than our estimate, the direction of association remains consistent, reinforcing the applicability of these findings across similar cultural and environmental contexts.

Cheng et al. in China proposed a cutoff of 20 ng/mL for predicting GDM risk based on early pregnancy vitamin D levels [25]. This is strikingly similar to our ROC-derived cutoff (<19.5 ng/mL), suggesting the possibility of a universal threshold for antenatal screening across Asian populations. Beyond epidemiological associations, mechanistic studies provide biological plausibility. Cho et al. reported altered placental expression of glucose transporters and inflammatory cytokines in vitamin D-deficient women with GDM [26]. While our study did not include placental biomarker analyses, such evidence strengthens the hypothesis that vitamin D influences glucose homeostasis not only via systemic insulin resistance but also through placental metabolic regulation. These results resonate strongly with our own, given shared cultural and environmental factors such as veiling practices and limited sun exposure. Taken together, our findings align with the majority of published literature, demonstrating a clear association between vitamin D deficiency and GDM. The novelty of our study lies in its trimester-specific design, robust biochemical analysis, and ROC methodology, which together provide strong evidence that first-trimester vitamin D levels may serve as a valuable predictor for GDM.

Despite the strengths of our study, several limitations must be acknowledged. First, this was a single-center study conducted in a tertiary care hospital in South India, which limits the generalizability of findings to broader and more diverse populations. Second, the sample size, though statistically adequate, was relatively small (n=87), reducing the power to explore subgroup analyses or detect modest associations. Third, as an observational study, causality cannot be inferred, and residual confounding from unmeasured variables such as calcium or parathyroid hormone levels cannot be excluded. Finally, vitamin D was measured only once in the first trimester; dynamic changes in maternal vitamin D status across pregnancy were not captured.

Despite these limitations, our study provides compelling evidence for incorporating vitamin D screening into routine antenatal care, especially in high-burden regions with widespread deficiency. The strong predictive value of early pregnancy vitamin D levels for GDM suggests that supplementation strategies could have dual benefits for maternal and fetal outcomes. Future research should include larger multicentric cohorts, interventional trials assessing supplementation efficacy, and mechanistic studies to better elucidate the pathways linking vitamin D to glucose metabolism and pregnancy outcomes.

## Conclusions

This prospective observational study demonstrated a strong and statistically significant association between first-trimester vitamin D deficiency and the subsequent development of GDM. Women with deficient vitamin D levels had a fivefold higher risk of developing GDM, and ROC analysis revealed excellent predictive accuracy for early pregnancy screening. The high prevalence of deficiency in our cohort, consistent with global and regional reports, underscores the urgent need for antenatal screening and timely supplementation as a low-cost, feasible strategy to reduce the burden of GDM and its complications. While further multicentric and interventional studies are warranted to confirm causality and establish standardized cut-off values, our findings provide compelling evidence to support early vitamin D assessment as an integral component of maternal health care.

## Appendices

**Table 5:** Case Record Form

Sl. No.	Variable	Details / Response Options
1	Participant ID	
2	Age (years)	
3	Height (cm)	
4	Weight (kg)	
5	Body Mass Index (BMI, kg/m²)	
6	Gestational Age at Recruitment (weeks)	
7	Parity	Primigravida/Multigravida
8	Socioeconomic Status	Upper/Middle/Lower (as per the modified Kuppuswamy scale)
9	Educational Status	Illiterate/Primary/Secondary/Graduate/Postgraduate
10	Occupation	Housewife/Employed/Self-employed/Other
11	Residence	Urban/Rural
12	Sunlight Exposure per day	<30 min / 30–60 min / >60 min
13	Physical Activity Level	Sedentary/Moderate/Active
14	Dietary Habits	Vegetarian/Mixed diet
15	Vitamin D Supplementation (prior to or during pregnancy)	Yes/No
16	Serum Vitamin D Level (ng/mL)	
17	Vitamin D Category	Deficient (<20 ng/mL) / Insufficient (20–30 ng/mL) / Sufficient (>30 ng/mL)
18	Fasting Blood Glucose (mg/dL)	
19	OGCT (2-hour value, mg/dL)	
20	Gestational Diabetes Mellitus (GDM) Diagnosis	Yes/No
21	Gestational Age at GDM Diagnosis (weeks)	
22	Family History of Diabetes Mellitus	Yes/No
23	Previous History of GDM or Macrosomia	Yes/No
24	Blood Pressure (mmHg)	
25	Hemoglobin (g/dL)	
26	Comments/Additional Notes	

## References

[1] Agarwal S, Kovilam O, Agrawal DK (2018). Vitamin D and its impact on maternal-fetal outcomes in pregnancy: a critical review. Crit Rev Food Sci Nutr.

[2] Wei SQ, Qi HP, Luo ZC, Fraser WD (2013). Maternal vitamin D status and adverse pregnancy outcomes: a systematic review and meta-analysis. J Matern Fetal Neonatal Med.

[3] Reverzani C, Zaake D, Nansubuga F, Ssempewo H, Manirakiza L, Kayiira A, Tumwine G (2025). Prevalence of vitamin D deficiency and its association with adverse obstetric outcomes among pregnant women in Uganda: a cross-sectional study. BMJ Open.

[4] Poel YH, Hummel P, Lips P, Stam F, van der Ploeg T, Simsek S (2012). Vitamin D and gestational diabetes: a systematic review and meta-analysis. Eur J Intern Med.

[5] Zhang MX, Pan GT, Guo JF, Li BY, Qin LQ, Zhang ZL (2015). Vitamin D deficiency increases the risk of gestational diabetes mellitus: a meta-analysis of observational studies. Nutrients.

[6] Pérez-López FR, Pasupuleti V, Mezones-Holguin E, Benites-Zapata VA, Thota P, Deshpande A, Hernandez AV (2015). Effect of vitamin D supplementation during pregnancy on maternal and neonatal outcomes: a systematic review and meta-analysis of randomized controlled trials. Fertil Steril.

[7] Burris HH, Rifas-Shiman SL, Kleinman K (2012). Vitamin D deficiency in pregnancy and gestational diabetes mellitus. Am J Obstet Gynecol.

[8] Fatima K, Asif M, Nihal K (2022). Association between vitamin D levels in early pregnancy and gestational diabetes mellitus: a systematic review and meta-analysis. J Family Med Prim Care.

[9] Lacroix M, Battista MC, Doyon M (2014). Lower vitamin D levels at first trimester are associated with higher risk of developing gestational diabetes mellitus. Acta Diabetol.

[10] Makgoba M, Nelson SM, Savvidou M, Messow CM, Nicolaides K, Sattar N (2011). First-trimester circulating 25-hydroxyvitamin D levels and development of gestational diabetes mellitus. Diabetes Care.

[11] Anand A, Mandal I, Hossain SB (2025). Prasad Scale 2025: an updated framework for socioeconomic assessment in India. Natl J Community Med.

[12] Tripathi R, Verma D, Gupta VK, Tyagi S, Kalaivani M, Ramji S, Mala YM (2017). Evaluation of 75 g glucose load in non-fasting state [Diabetes in Pregnancy Study group of India (DIPSI) criteria] as a diagnostic test for gestational diabetes mellitus. Indian J Med Res.

[13] Hu L, Zhang Y, Wang X (2018). Maternal vitamin D status and risk of gestational diabetes: a meta-analysis. Cell Physiol Biochem.

[14] Zhao R, Zhou L, Wang S, Yin H, Yang X, Hao L (2022). Effect of maternal vitamin D status on risk of adverse birth outcomes: a systematic review and dose-response meta-analysis of observational studies. Eur J Nutr.

[15] Song SJ, Si S, Liu J (2013). Vitamin D status in Chinese pregnant women and their newborns in Beijing and their relationships to birth size. Public Health Nutr.

[16] Palacios C, De-Regil LM, Lombardo LK, Peña-Rosas JP (2016). Vitamin D supplementation during pregnancy: updated meta-analysis on maternal outcomes. J Steroid Biochem Mol Biol.

[17] Bi WG, Nuyt AM, Weiler H, Leduc L, Santamaria C, Wei SQ (2018). Association between vitamin D supplementation during pregnancy and offspring growth, morbidity, and mortality: a systematic review and meta-analysis. JAMA Pediatr.

[18] Harvey NC, Holroyd C, Ntani G (2014). Vitamin D supplementation in pregnancy: a systematic review. Health Technol Assess.

[19] Tabrizi R, Moosazadeh M, Akbari M (2018). High prevalence of vitamin D deficiency among Iranian population: a systematic review and meta-analysis. Iran J Med Sci.

[20] Farrant HJ, Krishnaveni GV, Hill JC (2009). Vitamin D insufficiency is common in Indian mothers but is not associated with gestational diabetes or variation in newborn size. Eur J Clin Nutr.

[21] Agarwal MM, Dhatt GS, Shah SM (2010). Gestational diabetes mellitus: simplifying the international association of diabetes and pregnancy diagnostic algorithm using fasting plasma glucose. Diabetes Care.

[22] Luo C, Li Z, Lu Y (2022). Association of serum vitamin D status with gestational diabetes mellitus and other laboratory parameters in early pregnant women. BMC Pregnancy Childbirth.

[23] Deepa R, Schayck OC, Babu GR (2024). Low levels of vitamin D during pregnancy associated with gestational diabetes mellitus and low birth weight: results from the MAASTHI birth cohort. Front Nutr.

[24] Muthukrishnan J, Dhruv G (2015). Vitamin D status and gestational diabetes mellitus. Indian J Endocrinol Metab.

[25] Cheng Y, Chen J, Li T (2022). Maternal vitamin D status in early pregnancy and its association with gestational diabetes mellitus in Shanghai: a retrospective cohort study. BMC Pregnancy Childbirth.

[26] Cho GJ, Hong SC, Oh MJ, Kim HJ (2013). Vitamin D deficiency in gestational diabetes mellitus and the role of the placenta. Am J Obstet Gynecol.

